# The Predatory Stink Bug *Arma custos* (Hemiptera: Pentatomidae) Produces a Complex Proteinaceous Venom to Overcome Caterpillar Prey

**DOI:** 10.3390/biology12050691

**Published:** 2023-05-09

**Authors:** Yuli Qu, Andrew A. Walker, Ling Meng, Volker Herzig, Baoping Li

**Affiliations:** 1 Department of Entomology, School of Plant Protection, Nanjing Agricultural University, Nanjing 210095, China; 2018202040@njau.edu.cn (Y.Q.); ml@njau.edu.cn (L.M.); 2 Institute for Molecular Bioscience, The University of Queensland, Brisbane, QLD 4072, Australia; a.walker@imb.uq.edu.au; 3 School of Science, Technology and Engineering, University of the Sunshine Coast, Sippy Downs, QLD 4556, Australia; 4 Australian Research Council Centre of Excellence for Innovations in Peptide and Protein Science, Brisbane, QLD 4072, Australia; 5 Centre for Bioinnovation, University of the Sunshine Coast, Sippy Downs, QLD 4556, Australia

**Keywords:** predatory stink bugs, venom, protein, transcriptomic, proteomic, bioactivity, insecticidal, Asopinae, lepidoptera, caterpillars

## Abstract

**Simple Summary:**

We studied the salivary venom of a predatory stink bug, which captures prey by injecting venom through their specialized stylets. We aimed to understand the composition and function of the salivary venom, which had previously been poorly characterized. We collected gland extracts and venom samples from a stink bug and performed shotgun proteomic and transcriptomic analyses. The results showed that the salivary venom of the stink bug contained over 100 individual proteins, including various enzymes and proteins with different functions, but did not contain the proteins that were shared by and unique to other predatory heteropterans. The most abundant families of proteins in the salivary venom were hydrolase enzymes. We also found that the proteinaceous (>3 kDa) venom fraction of the stink bug had insecticidal activity against caterpillars. Our findings increase the understanding of salivary proteins in predatory stink bugs and suggest an innovative strategy of using these proteins to develop bioinsecticides.

**Abstract:**

Predatory stink bugs capture prey by injecting salivary venom from their venom glands using specialized stylets. Understanding venom function has been impeded by a scarcity of knowledge of their venom composition. We therefore examined the proteinaceous components of the salivary venom of the predatory stink bug *Arma custos* (Fabricius, 1794) (Hemiptera: Pentatomidae). We used gland extracts and venoms from fifth-instar nymphs or adult females to perform shotgun proteomics combined with venom gland transcriptomics. We found that the venom of *A. custos* comprised a complex suite of over a hundred individual proteins, including oxidoreductases, transferases, hydrolases, ligases, protease inhibitors, and recognition, transport and binding proteins. Besides the uncharacterized proteins, hydrolases such as venom serine proteases, cathepsins, phospholipase A_2_, phosphatases, nucleases, alpha-amylases, and chitinases constitute the most abundant protein families. However, salivary proteins shared by and unique to other predatory heteropterans were not detected in the *A. custos* venom. Injection of the proteinaceous (>3 kDa) venom fraction of *A. custos* gland extracts or venom into its prey, the larvae of the oriental armyworm *Mythimna separata* (Walker, 1865), revealed insecticidal activity against lepidopterans. Our data expand the knowledge of heteropteran salivary proteins and suggest predatory asopine bugs as a novel source for bioinsecticides.

## 1. Introduction

Predatory heteropterans are well-known as biocontrol agents for controlling pest populations in agricultural systems [1]. They feed by inserting their intricate needle-like stylets into their prey while releasing toxic saliva to paralyze prey and initiate extra-oral digestion [2,3]. The toxic saliva is considered venom, as it has been shown to be active in both vertebrates and invertebrates. For example, the venom of the red-spotted assassin bug *Platymeris rhadamanthus* leads to a rapid calcium influx in mammalian sensory neurons [4]. The venom of the predatory pentatomid bug *Podisus nigrispinus* is toxic to *Spodoptera frugiperda* caterpillars, causing damage to midgut cells resulting in apoptosis and necrosis [5]. The venom of the reduviid *Rhynocoris marginatus* results in the death of *Spodoptera litura* caterpillars when injected or when orally given in high doses, whereas lower oral doses lead to changes in physiology and digestive enzymes [6]. The venom of predatory heteropterans is produced by their salivary glands. It has been widely accepted that salivary glands, combined with piercing-sucking mouthparts, are modified into venom delivery apparatus [7]. The term “venom glands” has been used interchangeably with “salivary glands” in the recently existing literature, as the latter term seems to overlook their paralytic activity on prey. The venom gland of predatory heteropterans is a complex organ with paired structures, comprising the anterior lobes (AMG) and posterior lobes (PMG) of the main gland as well as the accessory gland (AG).

The identification of compounds in the salivary venom of predatory heteropterans is essential for assessing their potential for biological pest management. Recently, with the development of transcriptome and proteome analyses, the composition of predatory heteropteran venoms has been studied in several species, including assassin bugs (the family Reduviidae) [8,9], minute pirate bugs (the family Anthocoridae) [10], and true water bugs (the infraorder Nepomorpha) [11,12] (Figure S1). These studies revealed that venoms are typically complex cocktails of organic and inorganic compounds, with numerous proteinaceous components [13,14,15]. For instance, a plethora of digestive enzymes such as proteases, phospholipases, amylases, lipases, nucleases, and glucosidases have been detected in the predatory heteropteran venoms [16,17]. Additionally, the Ptu1 family of disulfide-rich peptides were identified in the venom of the assassin bug *Peirates turpis* and found to block calcium channels [18]. However, studies on the composition of salivary venom produced by predatory pentatomids (Hemiptera: Pentatomidae: Asopinae), especially their proteinaceous constituents, are limited. While lipases, alpha-amylases, and some proteases such as collagenase have been detected in predatory pentatomid venom gland extracts, their contribution to prey toxicity remains unclear [19,20,21,22]. To date, only two small molecule toxins, *N,N*-dimethylaniline and 1,2,5-trithiepane, have been confirmed to contribute to the insecticidal activity of the predatory stink bug *P. nigrispinus* [23].

The composition of predatory stink bug venom is also interesting from an evolutionary point of view. Previously, it has been reported that distantly related predaceous heteropterans such as belostomatid giant bugs (Nepomorpha) and the terrestrial reduviid assassin bugs (Cimicomorpha), whose ancestors maintained a predatory trophic strategy throughout their evolution, retain a suite of homologous venom proteins associated with predation, whereas groups that have shifted to trophic strategies of plant-feeding or blood-feeding have lost these predation-associated proteins and evolved different salivary compositions [11,12]. The predatory stink bugs, which are descended from the same group of predaceous heteropterans but switched to plant-feeding and then back to predation [24], are in a unique evolutionary position. It is uncertain if they will share the predation-associated heteropteran salivary proteins that are common to reduviids and belostomatids, or if these proteins were lost during the transition of their pentatomomorphan ancestors to plant feeding. If the latter, it is possible they recruited new salivary toxins de novo after their most recent transition back to a predatory trophic strategy.

**Figure 1:**
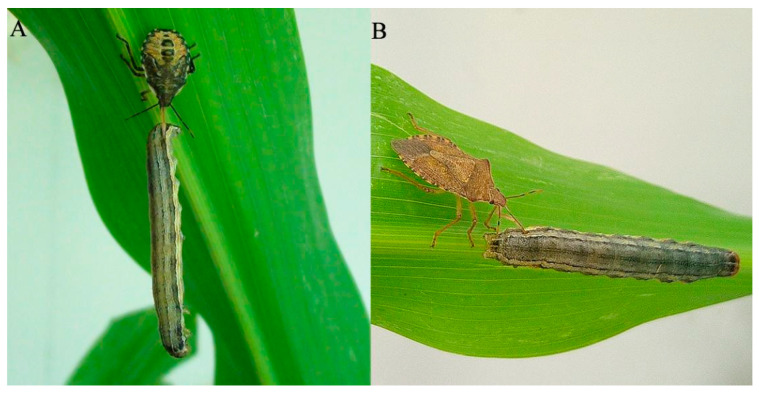
The stink bug *Arma custos* feeding on the oriental armyworm *Mythimna separata*. (**A**) A 5th-instar nymph feeding on a 6th-instar *M. separata* larva. (**B**) An adult female feeding on a 6th-instar *M. separata* larva.

The stink bug *Arma custos* (Fabricius, 1794) (=*Arma chinensis* (Fallou, 1881), the junior synonym [25]) is a generalist predator of crop pests, especially lepidopteran larvae (Figure 1). Similar to most predatory heteropterans, it employs a salivary venom to immobilize and kill its prey, even if the prey is larger than the bug itself. The time it takes for the prey to die varies depending on the size of the stink bug or the prey, ranging from a few minutes to several hours. However, paralysis occurs rapidly, usually within a few minutes (unpublished personal observations). To gain a holistic overview of the venom proteome of *A. custos*, we collected gland extracts and venom samples from fifth-instar nymphs or adult females using different methods and then utilized a combinatorial proteo-transcriptomic approach to elucidate the protein composition of *A. custos* venom. To investigate the biological effect of *A. custos* venom on prey insects, we injected oriental armyworms (*Mythimna separata*) with the collected gland extracts and venom samples. Our findings contribute to advancing our understanding of the predation capacity of the stink bug *A. custos* and provide a basis for the discovery of novel insecticidal compounds.

## 2. Materials and Methods

### 2.1. Insect Collection and Rearing

All insects were reared in the insectary at Nanjing Agricultural University at 26 ± 1 °C, 60 ± 10% relative humidity, and a 16:8 h light:dark photoperiod. The predatory stink bug *A. custos* was originally derived from the Biological Control Laboratory of the Institute of Plant Protection, Chinese Academy of Agricultural Sciences, in Beijing in 2017, and has since been maintained in the insectary at Nanjing Agricultural University. Eggs and the first-instar nymphs were kept in petri dishes (9 cm in diameter) with a piece of moistened cotton wool, while nymphs of other instars and adults were reared on a diet of *M. separata* larvae and fresh elm (*Ulmus pumila*) leaves. Adults were maintained in groups of 40–60 in 30 cm cubic cages covered with organza fabric and supplied with fresh elm stems for egg deposition.

The oriental armyworm *M. separata* was originally provided by the Research Institute of Agriculture and Forestry of Hebei province, China, in 2013, and has since been maintained in the insectary at Nanjing Agricultural University. Eggs were kept in petri dishes (9 cm in diameter) for hatching, and larvae were reared in groups of 40–60 in glass jars (9 cm in height and 20 cm in diameter) on a semi-synthetic artificial diet mainly consisting of maize leaves, wheat bran, sucrose, agar, yeast, and water [26]. Pupae were collected in groups of 40–60 in wooden frames and 30 cm cubic cages covered with organza fabric for adult emergence. Upon emergence, nylon ropes were suspended from the cage roof for egg deposition, and cotton balls soaked in a 20% (*v/v*) honey solution were provided as supplementary food for the moths.

### 2.2. Venom Collection and Concentration

We used two methods to harvest the venom from the 5th-instar stink bug nymphs. To extract venom directly by dissecting the venom glands, the nymphs were anesthetized on ice for 5 min and dissected under a dissecting stereomicroscope in a pre-cooled 0.1 M phosphate-buffered saline (PBS) solution. The dissected venom glands, as shown in Supplementary Figure S2, were immediately transferred to 1.5 mL microcentrifuge tubes containing 30 µL of PBS and then centrifuged at 5000× *g* for 1 min at 4 °C to empty the gland lumens. The glandular tissues were then removed with tweezers, and the crude homogenate was centrifuged at 15,000× *g* for 12 min at 4 °C, and the supernatant was transferred to a new tube. A total of 268 pairs of dissected venom glands were collected.

The second method for harvesting venom from 5th-instar stink bug nymphs was based on a non-invasive method known as sucrose solution diet feeding, which has been previously employed for collecting saliva from phytophagous hemipterans such as the brown planthopper *Nilaparvata lugens* [27], the Asian citrus psyllid *Diaphorina citri* [28], and the bean bug *Riptortus pedestris* [29]. We followed the method described by Huang et al. [27], with slight modifications. Briefly, a sterile diet with 2.5% sucrose was prepared and filtered through a 0.22 µm syringe filter (Millipore, Billerica, MA, USA) to eliminate microorganisms. Then, 2 mL of the sterile diet was contained between two layers of Parafilm^TM^ membrane (Bemis, Neenah, WI, USA) stretched over a sterile petri dish (9 cm in diameter). In each petri dish, there were six starved 5th-instar *A. custos* nymphs that were allowed to feed on the sterile diet for 24 h while releasing salivary venom in the meanwhile. After this period, the mixture of the sucrose solution and salivary venom was retrieved from the space between the parafilm layers with a pipette and transferred to a pre-cooled tube. A total of 700 nymphs were used for venom collection, with each being used only once.

To harvest the venom from adult stink bugs, we also used two methods. The first method was used to extract the venom directly by dissecting the venom glands of 207 adult stink bugs, including 101 females and 106 males. Additionally, we used a non-lethal electrostimulation method, as described by Walker et al. [30], with slight modifications, to harvest venom from 392 adult stink bugs (185 males and 207 females) one week after their final molt. Briefly, the stink bug was restrained on a foam platform with a rubber band, and its stylet was manually everted with a sterile pin. Clips connected to a power adaptor set to 24 V were placed on the thorax of the stink bug to induce the release of venom, which was then collected with a pipette and immediately transferred to a pre-cooled tube containing 20 μL of ultrapure water. The process was repeated until no more venom could be collected.

The gland extracts from *A. custos* 5th-instar nymphs (GE-n), venom samples from *A. custos* 5th-instar nymphs obtained by sucrose solution diet feeding method (VS-n), gland extracts from *A. custos* adults (GE-a), and venom samples from *A. custos* adults obtained by electrostimulation (VS-a) were concentrated using a 3 kDa molecular-weight-cutoff centrifugal filter (Millipore, Billerica, MA, USA) by centrifuging at 5000× *g* for 1 h at 4 °C. The concentrated samples were then immediately stored at −80 °C to prevent auto-degradation until further analysis.

### 2.3. Venom Gland Transcriptome Analysis

#### 2.3.1. RNA Extraction, cDNA Library Preparation and Illumina Sequencing

We collected 30 pairs of venom glands, as shown in Supplementary Figure S2, from 14 nymphs (5th instar), eight adult females, and eight adult males for RNA extraction. The collected glands were then ground in >10-fold the glandular volume of RNAlater solution (Beyotime, Shanghai, China) to prevent RNA degradation. Total RNA was extracted using the TRIzol Total RNA Isolation Kit (Takara, Dalian, China), according to the manufacturer’s instructions. The quantity and integrity of the extracted RNA were quantified using NanoDrop (NanoDrop Technologies, Wilmington, DE, USA) and the RNA Nano 6000 Assay Kit of the Bioanalyzer 2100 system (Agilent Technologies, Palo Alto, CA, USA), yielding a total of 30.98 μg and a quality score of 9.3, respectively. These results indicated that the RNA used for transcriptome sequencing was of high quality. The RNA-Seq libraries were constructed and sequenced by the Novogene company (Beijing, China). Briefly, poly-T oligo-attached magnetic beads were used to isolate polyadenylated mRNA from the total RNA, which was then fragmented using divalent cations at an elevated temperature in the First Strand Synthesis Reaction Buffer (5×). The first strand cDNA and the second strand cDNA were then synthesized. After end-repair and adaptor ligation, the products were PCR-amplified and purified using AMPure XP Beads (Beckman Coulter, Beverly, CA, USA) to generate the cDNA library, which was finally sequenced on an Illumina NovaSeq 6000 instrument.

#### 2.3.2. Transcriptome Assembly, Annotation, and Peptide Prediction

The image data measured by the high-throughput sequencer were converted into sequence data (FASTQ reads) by CASAVA base recognition. This process resulted in 19,781,556 bp of cleaned reads after removing reads containing adapter sequences, N base, and low-quality reads from the raw data. The cleaned reads were then assembled using Trinity v.2.6.6 software (http://trinityrnaseq.github.io, accessed 10 September 2021) to generate a total of 16,815 transcripts with a mean length of 1208 bp and an N50 of 1841 bp. The accuracy and completeness of the transcriptome were assessed using BUSCO v.3.1.0 software (https://gitlab.com/ezlab/busco, accessed 10 September 2021). The assembled unigenes were annotated using databases such as Nr, Swissprot, KEGG, and COG. For further protein identification of the proteome, an amino acid sequence library of the venom gland complex was generated by extracting all open reading frames (ORFs) translations via TransDecoder v.3.0.1 software (https://github.com/TransDecoder/TransDecoder/releases, accessed 10 September 2021).

### 2.4. Venom Proteome Analysis

**Figure 2:**
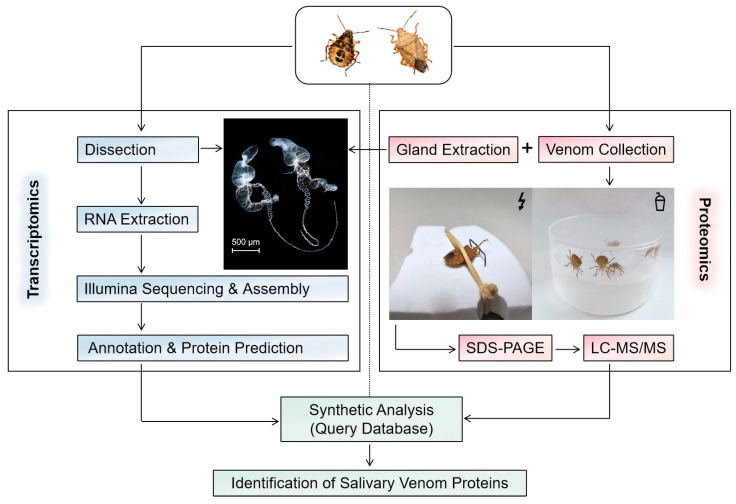
Schematic workflow of using an integrated transcriptomic and proteomic analyses to identify proteins of the salivary venom from the stink bug *Arma custos.*.

The protein identification and quantification were performed with shotgun proteomics (Figure 2) by the Shanghai Applied Protein Technology Company (Shanghai, China). The protein components were initially separated by sodium dodecyl sulfate–polyacrylamide gel electrophoresis (SDS-PAGE) and visualized using Coomassie blue staining. A total of 20 µL of each sample was loaded, with approximate amounts of 9.1 µg for VS-n, 24.7 µg for GE-n, 27.5 µg for GE-a, and 9.3 µg for VS-a. Then samples underwent an enzymolytic process through the filter-aided sample preparation (FASP) method. Briefly, each sample (20 µL) was diluted with dithiothreitol (DTT) to a final concentration of 100 mM, subjected to a boiling water bath for 5 min, and then cooled to room temperature (RT). After adding 200 µL of urea (UA) buffer, samples were filtered with a 30 kDa ultrafiltration centrifuge tube by centrifuging at 14,000× *g* for 15 min, and filtrates were discarded. This step was repeated once. The samples were then alkylated with 100 µL of iodoacetamide (IAA) buffer (100 mM IAA in UA buffer) by vortexing at 600 rpm for 1 min, incubated at RT in the absence of light for 30 min, and centrifuged again at 14,000× *g* for 15 min. The trapped proteins on the filter were washed twice with 100 µL of UA buffer by centrifuging at 14,000 *g* for 15 min and twice with 100 µL of 25 mM NH_4_HCO_3_ solution by centrifuging at 14,000× *g* for 15 min. Proteins were digested with 40 µL of trypsin buffer (3 ug of trypsin in 40 µL of 100 mM NH_4_HCO_3_ solution) by vortexing at 600 rpm for 1 min and incubated at 37 °C for 16–18 h. The samples were then transferred to new collection tubes and centrifuged at 14,000× *g* for 15 min. Finally, the digested proteins were collected by centrifuging at 14,000× *g* for 15 min after adding 40 µL of 25 mM NH_4_HCO_3_ solution. The peptides were then desalted on C18 cartridges, redissolved in 40 µL of 0.1% formic acid solution after being lyophilized, and quantified for their OD_280_.

The enzymatic products were then taken for liquid chromatography–mass spectrometry/mass spectrometry (LC-MS/MS) analysis. Briefly, the peptide digests were separated by a high-performance liquid-chromatography system, Easy nLC (Thermo Scientific, Waltham, MA, USA). Solvent A (0.1% formic acid solution) and solvent B (84% acetonitrile and 0.1% formic acid solution) were used as the mobile phases for gradient separation. The sample was loaded onto the chromatographic column (Thermo Scientific EASY column, 2 cm long, 100 μm inner diameter, 5 μm C18) which was balanced with 95% solvent A, and then separated by an analytical column (Thermo Scientific EASY column, 10 cm long, 75 μm inner diameter, 3 μm C18) at a constant flow rate of 300 nL/min. The following gradient was applied to perform separation: the linear gradient of solvent B ranged from 0 to 35% over 0–50 min, then 35% to 100% over 50–55 min, and then held at 100% for 5 min. The MS data were analyzed for 60 min with a Q-Exactive mass spectrometer (Thermo Scientific, Waltham, MA, USA). To acquire the full MS scan, the scanning range of the parent ions was set to 300–1800 *m/z* with the positive ion mode as a detection method. Twenty fragment patterns (MS/MS scan) were then collected from the full MS scan to determine the mass–charge ratio of polypeptides and polypeptide fragments using higher-energy collision dissociation (HCD) activation. The resolution and maximum injection time were set to 70,000 (at *m/z* 200) and 50 ms for the full MS and 17,500 (at *m/z* 200) and 60 ms for MS/MS, respectively. The automatic gain control (AGC) target was set to 3 × 10^6^. The number of microscans, isolation window, normalized collision energy (NCE), and dynamic exclusion duration were set to 1, 2 *m/z*, 27 eV, and 60 s, respectively. The underfill ratio, which specifies the minimum percentage of the target value likely to be reached at maximum fill time, was defined as 0.1%.

### 2.5. Injection of Proteinaceous Venom in M. separata Larvae

The biological effects of proteinaceous (>3 kDa) venom were investigated by injection into 3rd-instar *M. separata* larvae with an average body weight of 78.75 mg (ranging from 50–100 mg). The protein concentrations of GE-n, VS-n, GE-a, and VS-a were initially determined following the protocol of a Bradford protein concentration assay kit (Beijing Solarbio Science & Technology Company, Beijing, China). Based on absorbance values at 595 nm, the measured protein concentrations were 1.233, 0.458, 1.375, and 0.466 μg/μL, respectively. All samples were then diluted to the same concentration of 0.458 μg/μL. Then 3 µL of the proteinaceous venom sample was injected into individual *M. separata* larvae that had been anesthetized for 5 min over ice. Injection was performed subcutaneously in the thorax region using a 33 G needle on a 10 μL microsyringe (Hamilton, Reno, NV, USA). Larvae were injected with an equal volume of PBS, sucrose solution, or ultrapure water as the control for each protein solution, respectively. Thereafter, the larvae were placed individually in Petri dishes (9 cm in diameter) and fed with the artificial diet. The survival of the larvae was monitored daily for up to 5 days. Twenty *M. separata* larvae were injected for each group.

### 2.6. Data Analysis

For protein identification, the MS/MS-spectrum data were searched using MaxQuant v.1.5.1.0 software (https://maxquant.org/maxquant/, accessed 10 November 2021) against the deduced amino acid sequence library translated from the glandular transcriptome. The parameters for database searching included trypsin as the proteolytic enzyme for cleavage, allowance for two missed cleavage sites, carbamidomethyl as a fixed modification, and oxidation (M) and acetyl (Protein N-term) as variable modifications. The false discovery rate (FDR) was also estimated. Peptides were filtered at a 1% FDR at the peptide spectrum match level. Then the identified proteins were classified based on their annotation results in the Nr database. The homology searches of the proteins in *A. custos* venom against venom proteins in the assassin bug *Pristhesancus plagipennis* were performed using Blast search. The relative quantity of each protein in the different samples was quantified using the label-free quantification (LFQ) value calculated by MaxQuant.

To analyze the data about the effect of gland extracts and venom samples from *A. custos* on the survival of *M. separata* larvae, we employed the R package *survival* [31] to determine the survival probabilities and compare them with the log-rank test. Survival curves were plotted with the R package *survminer* [32]. The statistical analyses were carried out using RStudio v.1.4.1717 [33].

## 3. Results

### 3.1. Protein Components of A. custos Salivary Venom

**Figure 3:**
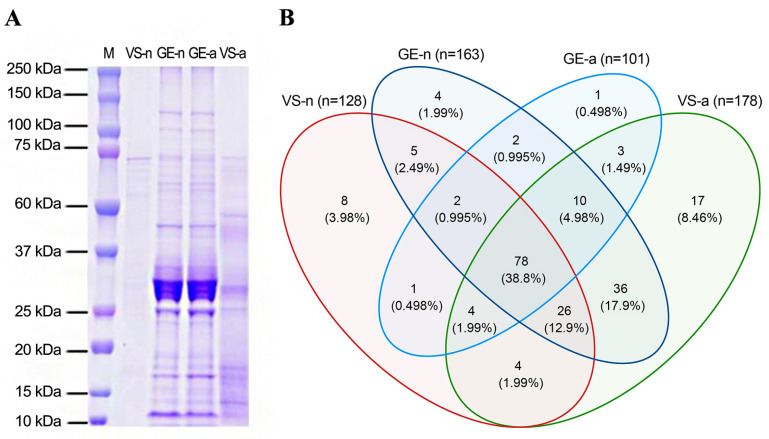
SDS-PAGE analysis and Venn diagram comparisons of *Arma custos* venom proteins. (**A**) Protein separation from gland extracts and venom samples of 5th-instar nymphs or adults on a Coomassie stained gel. The molecular weights for the protein marker are indicated on the left. (**B**) Venn diagrams indicating the numbers of proteins identified in gland extracts and venom samples from 5th-instar nymphs or adults. M: protein marker; VS-n: venom samples from 5th-instar nymphs obtained by sucrose solution diet feeding method; GE-n: gland extracts from 5th-instar nymphs; GE-a: gland extracts from adults; VS-a: venom samples from adults obtained by electrostimulation.

**Figure 4:**
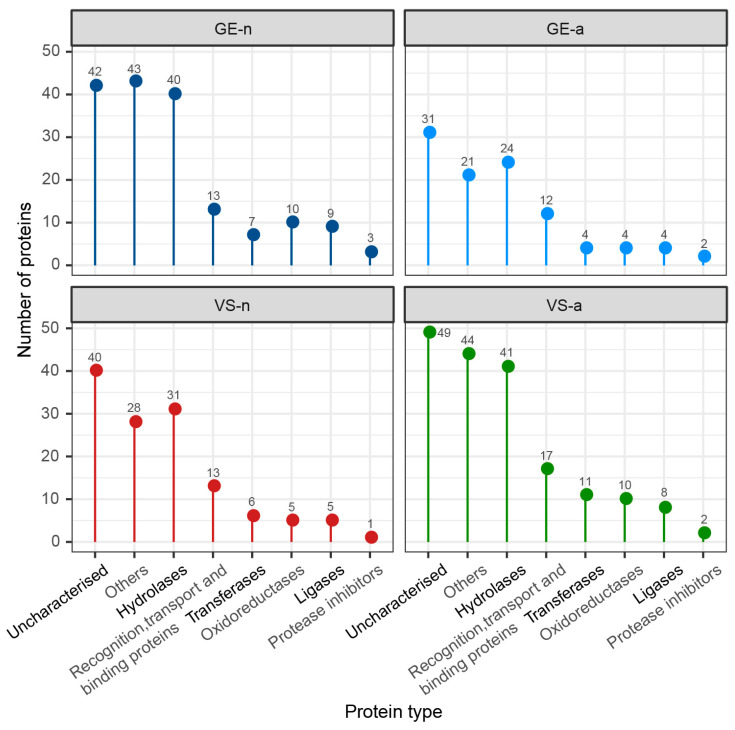
Number of different protein types in *Arma custos* gland extracts and venom samples from 5th-instar nymphs or adults. GE-n: gland extracts from 5th-instar nymphs; GE-a: gland extracts from adults; VS-n: venom samples from 5th-instar nymphs obtained by sucrose solution diet feeding method; VS-a: venom samples from adults obtained by electrostimulation.

**Figure 5:**
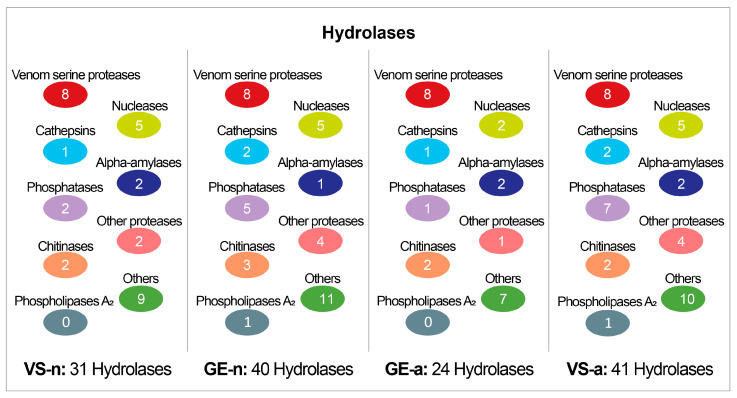
Number of different types of hydrolases in *Arma custos* gland extracts and venom samples from 5th-instar nymphs or adults. VS-n: venom samples from 5th-instar nymphs obtained by sucrose solution diet feeding method; GE-n: gland extracts from 5th-instar nymphs; GE-a: gland extracts from adults; VS-a: venom samples from adults obtained by electrostimulation.

The SDS-PAGE revealed abundant proteins in gland extracts and venom samples from both the fifth-instar nymphs and adults of *A. custos* (Figure 3A). The protein bands of the fifth-instar nymph venom samples were less clearly compared to the others. The molecular weights of proteins in gland extracts range from 11 kDa up to 230 kDa, with the most abundant being in the range of 10 to 35 kDa, which were similar to those found in adult venom samples. Shotgun LC-MS/MS analysis revealed that 163, 128, 101, and 178 proteins were identified in GE-n, VS-n, GE-a, and VS-a, respectively (Figure 3B). According to the annotation results of identified proteins in the Nr database, proteins in *A. custos* salivary venom were classified into the following eight groups: oxidoreductases; transferases; hydrolases; ligases; protease inhibitors; recognition, transport, and binding proteins; other proteins; and uncharacterized proteins (Figure 4). The majority of characterized proteins were hydrolases, including venom serine proteases, cathepsins, phospholipase A_2_ (PLA_2_), phosphatases, nucleases, alpha-amylases, and chitinases, among others (Figure 5). Notably, Blast search showed that only a part of *A. custos* salivary proteins (29 or 22.66% of VS-n proteins, 29 or 17.79% of GE-n proteins, 23 or 22.77% of GE-a proteins, and 27 or 15.17% of VS-a proteins, respectively) are homologous to *P. plagipennis* venom proteins, with all of these proteins occurring broadly across the tree of life rather than being unique to predatory heteropteran venoms (with the single exception of heteropteran venom family 12). The list of the proteins detected in *A. custos* venom is presented in Supplementary Table S1 with detailed information.

**Figure 6:**
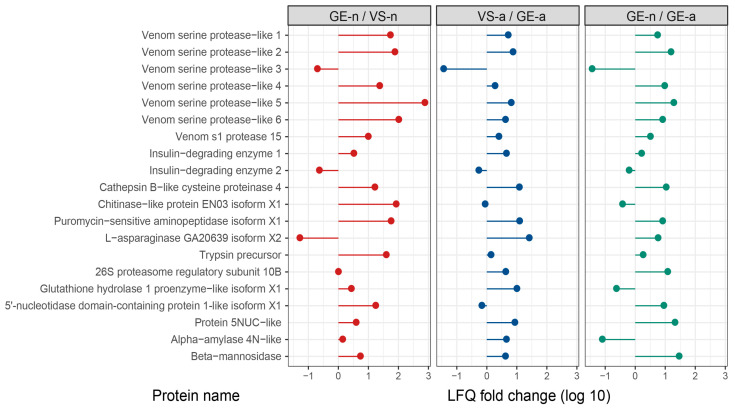
Comparison of LFQ intensity values for some major venom proteins in *Arma custos* gland extracts and venom samples from 5th-instar nymphs or adults. VS-n: venom samples from 5th-instar nymphs obtained by sucrose solution diet feeding method; GE-n: gland extracts from 5th-instar nymphs; GE-a: gland extracts from adults; VS-a: venom samples from adults obtained by electrostimulation.

Supplementary Tables S2–S5 list the top 20 most abundant proteins in gland extracts and venom samples of *A. custos* fifth-instar nymphs and adults, ranked by LFQ intensity values. We compared the protein abundance of some major venom proteins such as venom serine proteases, venom s1 protease, insulin-degrading enzymes, cathepsin B-like cysteine proteinase, chitinase-like protein, etc., in gland extracts and venom samples from the fifth-instar nymphs or adults. We found that in the fifth-instar nymphs, the abundance of these proteins was higher in gland extracts than in venom samples overall, whereas in adults, the opposite was observed. Moreover, the abundance of these proteins in the fifth-instar nymph gland extracts was higher, compared to adult gland extracts overall (Figure 6).

### 3.2. Proteinaceous Venom Activity against M. separata Larvae

**Figure 7:**
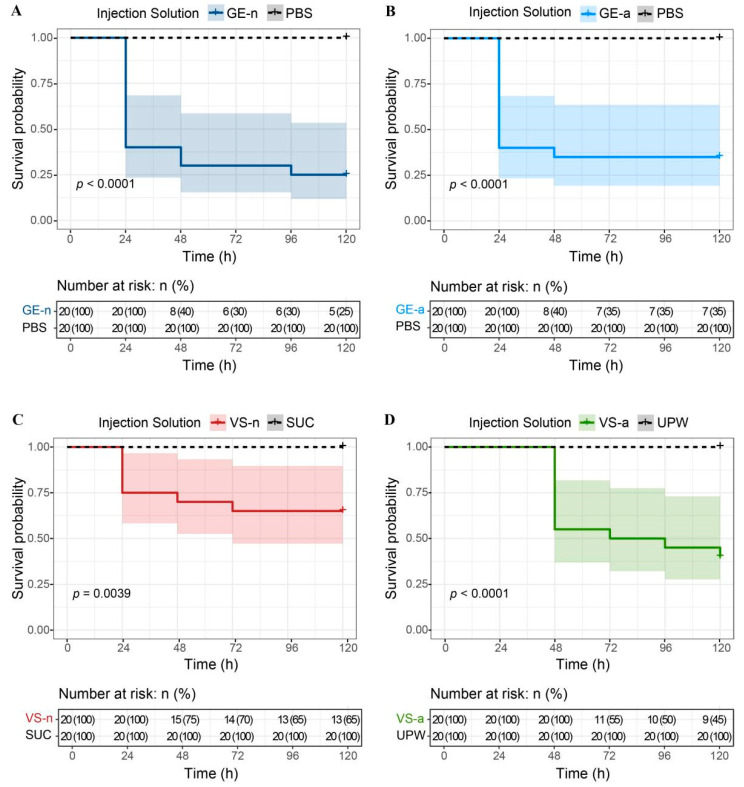
Effects of *Arma custos* gland extracts and venom samples from 5th-instar nymphs or adults on the survival of *Mythimna separata* larvae. (**A**) The survival probability of *M. separata* larvae injected with GE-n or PBS. (**B**) The survival probability of *M. separata* larvae injected with GE-a or PBS. (**C**) The survival probability of *M. separata* larvae injected with VS-n or SUC. (**D**) The survival probability of *M. separata* larvae injected with VS-a or UPW. GE-n: gland extracts from 5th-instar nymphs; PBS: phosphate-buffered saline; GE-a: gland extracts from adults; VS-n: venom samples from 5th-instar nymphs obtained by sucrose solution diet feeding method; SUC: sucrose solution; VS-a: venom samples from adults obtained by electrostimulation; UPW: ultrapure water. Shaded areas represent the 95% confidence intervals.

We found that the survival probability of *M. separata* larvae injected with GE-n, GE-a, VS-n, and VS-a significantly decreased at 5 days by 75% (*χ*^2^ = 24.7, *df* = 1, *p* < 0.01), 65% (*χ*^2^ = 19.1, *df* = 1, *p* < 0.01), 35% (*χ*^2^ = 8.3, *df* = 1, *p* < 0.01), and 60% (*χ*^2^ = 17.2, *df* = 1, *p* < 0.01), compared to controls, respectively. None of the control injections (PBS, sucrose solution, or ultrapure water) resulted in any death of *M. separata* larvae within 5 days (Figure 7). Moreover, GE-n showed a more lethal effect on larvae, compared to VS-n (*χ*^2^ = 6.6, *df* = 1, *p* = 0.01). Although there was no statistically significant difference between GE-a and VS-a on the survival probability of larvae (*χ*^2^ = 1.99, *df* = 1, *p* = 0.16), the insecticidal effect of the latter had a slower on-set, as all larvae treated with VS-a survived for at least 24 h. No significant difference was observed between the survival probabilities of larvae treated with GE-n and GE-a (*χ*^2^ = 0.32, *df* = 1, *p* = 0.57) (Table 1).

## 4. Discussion

In the current study, we utilized three distinct methods for harvesting venom from the fifth-instar nymphs and adults of *A. custos*, revealing similar but different protein compositions and effects on *M. separata* larvae. Gland extracts displayed different SDS-PAGE profiles, compared to venom samples obtained either by electrostimulation or the artificial feeding protocol. Gland extracts from the fifth-instar nymphs showed a more lethal effect on *M. separata* larvae, compared to their venom samples collected using the sucrose solution diet feeding method. This difference may be explained by a higher abundance of some major venom proteins in gland extracts overall, as indicated by the LFQ intensity value. Venom samples from *A. custos* adults obtained by electrostimulation had a slower onset of the lethal effect on *M. separata* larvae, compared to their gland extracts, which might indicate that venom obtained by electrostimulation contains more paralyzing toxins and less lethal toxins. This is possible with the actual venom. After all, in a natural setting, the prey is not immediately killed by the venom after being captured but rather dies slowly during the feeding process. However, electrostimulation is not a physiologically relevant stimulus and might give different results between species, considering that it may induce both release of gland contents by contraction of basal membranes or retention of gland contents through tightening of muscle-controlled valves. Several studies have confirmed that the method of harvesting venom from predatory heteropterans affects the composition and concentration of the venom and is linked to the functional compartmentalization of venom glands. In the case of the assassin bug *P. plagipennis*, venom collected through electrostimulation resulted in instant paralysis and death when injected into crickets, while the venom obtained through harassment did not. This is because venom collected through electrostimulation originated from the posterior main gland, which produces offensive venom that effectively paralyzes and kills prey insects, whereas venom collected through harassment originated from the anterior main gland, which produces a different venom that has a much milder effect on prey [34]. The functional compartmentalization of venom glands has also been confirmed in assassin bugs *Platymeris biguttatus* and *Psytalla horrida*, and the predatory stink bug *Brontocoris tabidus* [22,35,36]. It would therefore be intriguing to examine the anatomy of the venom gland in the stink bug *A. custos* for the presence of different compartments and for any potential differences in protein expression among them, as well as the glandular origin of bioactive venoms obtained through different methods.

The venoms of predatory heteropterans are known to be enriched with enzymes, peptides, and proteins, with hydrolases making up a significant portion, and *A. custos* venom contains many of the protein families associated with predatory heteropteran venoms (Table 2). Although hydrolases are primarily involved in the extra-oral digestion process, there is also evidence suggesting that they contribute to the toxicity of the venom on the prey [37]. Our transcriptome and proteome analyses indicated the presence of a plethora of hydrolases in venom produced by the stink bug *A. custos*, such as venom serine proteases, cathepsins, PLA_2_, phosphatases, nucleases, alpha-amylases, and chitinases, among others. These results support previous findings on the proteinaceous components in predatory heteropteran venoms. For instance, venom serine proteases, which may arise from venom self-digestion and are responsible for the proteolysis, have been detected at significantly high expression levels in the venom glands of the assassin bug *P. plagipennis*, the water scorpion *Laccotrephes japonensis*, and the stink bug *Andrallus spinidens* [21,38,39]. Cathepsin, another major enzymatic component found in predatory heteropterans such as the predatory stink bug *Podisus maculiventris*, may assist in breaking down complex macromolecules [40]. PLA_2_ plays a role in the digestion and emulsification of dietary lipids and has been detected in the venom of the giant water bug *Belostoma anurum* [13,41]. Phosphatases, found in the venom glands of the stink bug *P. nigrispinus*, are involved in the hydrolysis of organic phosphate esters [42]. Alpha-amylase is used by zoophytophagous heteropterans such as the big-eyed bug *Geocoris punctipes* and the stink bug *B. tabidus* to digest both plant-derived starch and prey-derived glycogen [19,22,43]. Chitinases, a group of chitin-degrading glycosidase, have also been found in the venom of some heteropteran predators, such as the assassin bug *Rhynocoris iracundus*, and may play a vital role in insecticidal and cytolytic activities [8,44]. In addition to hydrolases, there are other proteins in the venom produced by stink bug *A. custos* that might also contribute to its potency. For example, our study discovered transferrin in *A. custos* venom, which has been shown to be abundant in the accessory glands extract of the twin-spotted assassin bug *P. biguttatus* and has been hypothesized to help bugs avoid infection with bacterial pathogens of the captured prey during feeding [36,45].

On the other hand, the venom of *A. custos* lacks (with a single exception, see Table 2) other families of proteins that occur in predatory heteropteran venoms such as families 1, 2, 3, 5, and 10, which are conserved in heteropterans such as reduviids and belostomatids that diverged anciently but likely maintained predation constant trophic strategy [11,12]. Most likely, these protein families were either lost or repurposed during the transition of their pentatomomorphan ancestors to plant-feeding. The high abundance of many unknown and uncharacterized proteins in *A. custos* venom suggests that the predatory stink bugs have recruited venom toxins from multifunctional classes of proteins, such as hydrolase enzymes that were retained through trophic transition. Furthermore, they presumably recruited and developed their own unique toxin families within the Asopinae. The role of these uncharacterized proteins and unknown proteins in *A. custos* venom merits further research.

Our finding of a significant mortality in *M. separata* larvae when injected with proteinaceous gland extracts or venom samples from the stink bug *A. custos* is consistent with the existing research indicating that proteinaceous compounds in the venom of predatory heteropterans are responsible for the lethal effect on prey [7]. However, it was also reported that the toxic effects of the venom of heteropteran predators are due to small molecules. For example, non-proteinaceous salivary compounds such as *N,N*-dimethylaniline and 1,2,5-trithiepane, which are found in the venom of the predatory stink bug *P. nigrispinus* have been shown to have insecticidal effects [23]. It would therefore be interesting to investigate whether insecticidal small molecules are also present in the salivary venom of *A. custos*.

## 5. Conclusions

In the current study, we found that the salivary venom of *A. custos* comprised a complex suite of over a hundred individual proteins, of which hydrolases constitute the most abundant protein families. However, the salivary proteins that are shared by and unique to other predatory heteropterans were not detected in *A. custos* venom. In addition, we found that the proteinaceous (>3 kDa) venom fraction is responsible for the insecticidal activity against the oriental armyworm *M. separata*. Our findings increase the understanding of venom proteins in predatory heteropterans and provide a basis for the discovery of novel insecticidal compounds. Further research is needed to examine the anatomy of *A. custos* venom glands for any functional specializations, to assess the key protein components responsible for the insecticidal effects, and to explore insecticidal small molecules in the salivary venom of *A. custos*.

## Figures and Tables

**Table 1:** The results of log-rank tests for comparing survival curves of *Mythimna separata* larvae treated by *Arma custos* gland extracts and venom samples from 5th-instar nymphs or adults. VS-n: venom samples from 5th-instar nymphs obtained by sucrose solution diet feeding method; GE-n: gland extracts from 5th-instar nymphs; VS-a: venom samples from adults obtained by electrostimulation; GE-a: gland extracts from adults.

Comparison	Injection Solution	N	Observed	Expected	(O-E)^2^/E	(O-E)^2^/V	*χ* ^2^	*df*	*p*
VS-n vs. GE-n	VS-n	20	7	11.80	1.98	6.60	6.60	1	0.01
	GE-n	20	15	10.20	2.31	6.60			
VS-a vs. GE-a	VS-a	20	12	14.90	0.57	1.99	1.99	1	0.16
	GE-a	20	13	10.10	0.84	1.99			
GE-n vs. GE-a	GE-n	20	15	14.00	0.08	0.32	0.32	1	0.57
	GE-a	20	13	14.00	0.08	0.32			

**Table 2:** Major saliva and venom proteins reported in heteropteran insects.

Trophic Strategy	Polypeptide Family	Detected in Families	Detected in Lineage with Trophic Strategy	Activity	Citation
All trophic strategies	Proteases	Pentatomidae, Reduviidae, Belostomatidae, Nepidae, Naucoridae, Notonectidae, Corixidae, Coreidae	Predatory and herbivorous heteropterans	Protein catabolism	[4,11,12,38,39,46], this study
	Amylase/glycogenase	Pentatomidae, Reduviidae, Belostomatidae, Nepidae	Predatory and herbivorous heteropterans	Starch catabolism	[11,12,46,47], this study
	Lipase	Pentatomidae, Reduviidae, Belostomatidae, Nepidae, Naucoridae, Notonectidae, Corixidae, Coreidae	Predatory and herbivorous heteropterans	Lipid catabolism	[11,12,38,39], this study
	Cystatin	Pentatomidae, Reduviidae, Belostomatidae, Nepidae, Cimicidae, Coreidae	Predatory, herbivorous, and blood-feeding heteropterans	Protease inhibition	[11,38,39]
	Apolipophorin	Pentatomidae, Reduviidae, Belostomatidae, Nepidae, Cimicidae, Coreidae	Predatory, herbivorous, and blood-feeding heteropterans	Lipid binding	[11,12,38,39,46], this study
Predation- associated	CUB domain only	Reduviidae, Belostomatidae, Nepidae, Anthocoridae	Predatory heteropterans outside Pentatomomorpha	Paralytic?	[9,10,11,12,39]
	ICK-like peptides	Reduviidae, Naucoridae	Predatory heteropterans outside Pentatomomorpha	Paralytic?	[12,48]
	Haemolysin-like	Reduviidae, Belostomatidae, Nepidae	Predatory heteropterans outside Pentatomomorpha	Unknown	[12,34]
	Redulysin	Reduviidae	Predatory and blood-feeding heteropterans outside Pentatomomorpha	Anti- bacterial?	[4,38,39,49]
	Venom Family 1, 3, 5, 10	Reduviidae, Belostomatidae, Naucoridae, Notonectidae, Nepidae	Predatory heteropterans outside Pentatomomorpha	Unknown	[11,12,38,39]
	Venom Family 2	Reduviidae, Belostomatidae, Nepidae, Naucoridae, Notonectidae	Predatory heteropterans outside Pentatomomorpha	Paralytic?	[4,12,38,39,49]
	Family 12	Pentatomidae: Asopinae, Reduviidae, Belostomatidae, Nepidae	Predatory heteropterans	Unknown	[11,12,39], this study
	Other proteins in Suppl. Table S1	Pentatomidae: Asopinae	Predatory Pentatomomorpha: *Arma custos*	Unknown	this study
Haematophagy-associated	Triabin	Reduviidae, especially Triatominae	Blood-feeding heteropterans	Numerous	[39,50]
	Apyrase	Triatominae, Cimicidae	Blood-feeding heteropterans	Inhibition of platelet aggregation	[50,51]

## Data Availability

The transcript sequences of *A. custos* salivary glands were submitted to the National Center for Biotechnology Information (NCBI) Sequence Read Archive database with the accession number of PRJNA935089. The mass spectrometry proteomics data have been deposited to the ProteomeXchange Consortium via the PRIDE partner repository (http://www.ebi.ac.uk/pride/, accessed 6 February 2023) with the dataset identifier PXD040272. For reviewer access, please use the following username and password: username—reviewer_pxd040272@ebi.ac.uk, password—l3NASi7w.

## Supplementary Materials

The following supporting information can be downloaded at https://www.mdpi.com/article/10.3390/biology12050691/s1, Figure S1: Phylogram of Heteroptera, depicting the superfamily-level relationships and listing the species of which the venoms have already been studied. Phylogenies modified and simplified from Johnson and colleagues [52], Wang and colleagues [53], and Li and colleagues [54]; Figure S2: Venom glands of *Arma custos* 5th-instar nymphs. (A) Anterior lobes of the main gland. (B) Posterior lobes of the main gland. (C) Accessory glands. Scale bar is shown at the bottom left corner of the image. Table S1: The identification and quantification results of proteins and peptides in the stink bug *Arma custos* venom detected by LC-MS/MS (Sheet 1: Protein Summary; Sheet 2: Peptide Summary); Table S2: Top 20 proteins in terms of the LFQ intensity value in venom samples from *Arma custos* 5th-instar nymphs obtained by sucrose solution diet feeding method (VS-n); Table S3: Top 20 proteins in terms of the LFQ intensity value in gland extracts from *Arma custos* 5th-instar nymphs (GE-n); Table S4: Top 20 proteins in terms of the LFQ intensity value in gland extracts from *Arma custos* adults (GE-a); Table S5: Top 20 proteins in terms of the LFQ intensity value in venom samples from *Arma custos* adults obtained by electrostimulation (VS-a).
