# Supplementary material for: The Predatory Stink Bug Arma custos (Hemiptera: Pentatomidae) Produces a Complex Proteinaceous Venom to Overcome Caterpillar Prey
Source: Biology (Basel). 2023 May 9;12(5):691. doi: 10.3390/biology12050691 (PMC10215959; doi:10.3390/biology12050691)
Supplement: Supplementary file 1 [file biology-12-00691-s001.zip › biology-2352946-Supplementary.pdf]

Supplementary

The Predatory Stink Bug *Arma custos* (Hemiptera: Pentatomidae) Produces a Complex Proteinaceous Venom to Overcome Caterpillar Prey

Yuli Qu [1,2,3], Andrew A. Walker [2,4], Ling Meng [1], Volker Herzig [2,3,5,*] and Baoping Li [1,*]

1 Department of Entomology, School of Plant Protection, Nanjing Agricultural University, Nanjing 210095, China; 2018202040@njau.edu.cn (Y.Q.); ml@njau.edu.cn (L.M.)
2 Institute for Molecular Bioscience, The University of Queensland, Brisbane, QLD 4072, Australia; a.walker@imb.uq.edu.au
3 School of Science, Technology and Engineering, University of the Sunshine Coast, Sippy Downs, QLD 4556, Australia
4 Australian Research Council Centre of Excellence for Innovations in Peptide and Protein Science, Brisbane, QLD 4072, Australia
5 Centre for Bioinnovation, University of the Sunshine Coast, Sippy Downs, QLD 4556, Australia
* Correspondence: vherzig@usc.edu.au (V.H.); lbp@njau.edu.cn (B.L.)

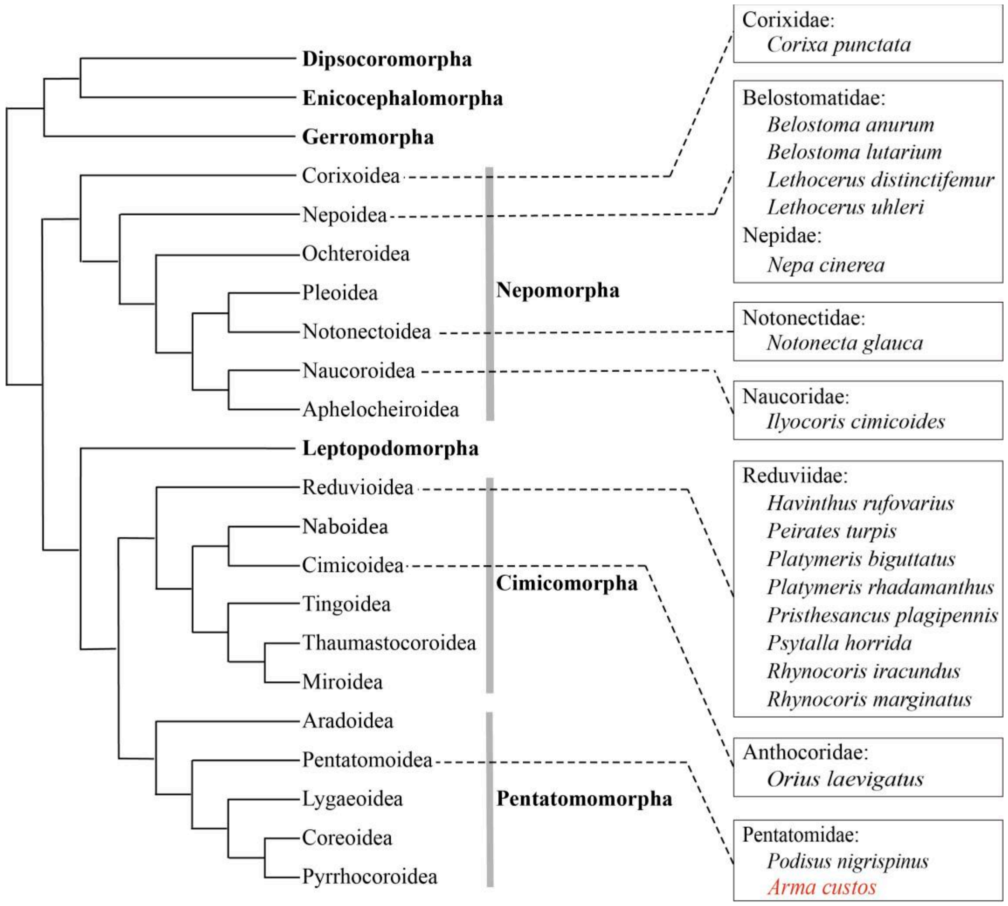

Supplementary Figure S1: Phylogram of Heteroptera, depicting the superfamily-level relationships and listing the species of which the venoms have already been studied. Phylogenies modified and simplified from Johnson and colleagues [52], Wang and colleagues [53], and Li and colleagues [54].

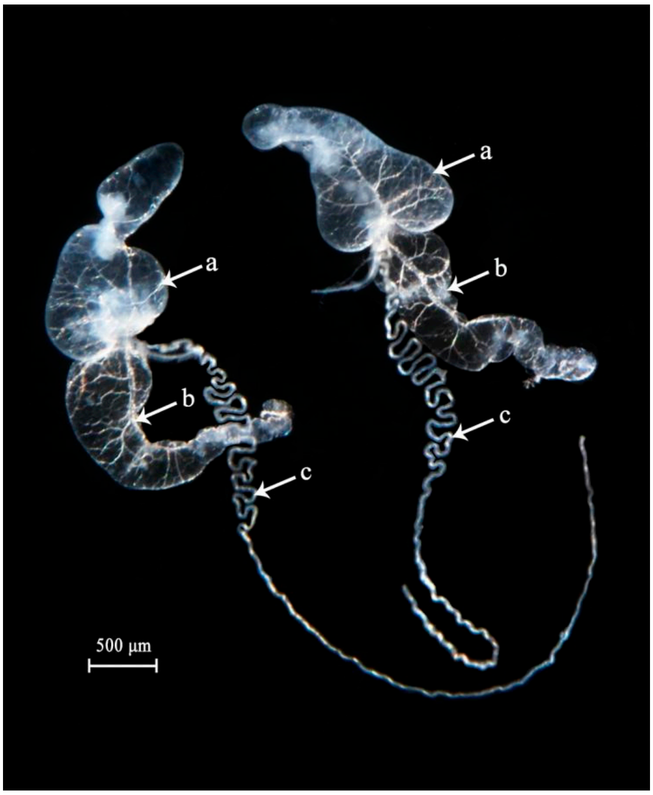

Supplementary Figure S2: Venom glands of *Arma custos* 5th-instar nymphs. **(a)** Anterior lobes of the main gland. (**b**) Posterior lobes of the main gland. (**c**) Accessory glands. Scale bar is shown at the bottom left corner of the image.

Supplementary Table S1 (see Excel file): The identification and quantification results of proteins and peptides in the stink bug *Arma custos* venom detected by LC-MS/MS (Sheet 1: Protein Summary; Sheet 2: Peptide Summary);

Supplementary Table S2: Top 20 proteins in terms of the LFQ intensity value in venom samples from *Arma custos* 5th-instar nymphs obtained by sucrose solution diet-feeding method (VS-n).

Number	Protein	LFQ Intensity
1	LOW QUALITY PROTEIN: insulin-degrading enzyme ②	14852000
2	protein turtle isoform X4	9182400
3	odorant-binding protein 18	2108900
4	membrane-spanning 4-domains subfamily A member 14-like	1768200
5	unknown protein ③	1590200
6	transferrin-like	1369200
7	tropomyosin-1, isoforms 9A/A/B isoform X15	1236400
8	heat shock 70 kDa protein 4	1209200
9	unknown protein ⑯	1031600
10	unknown protein ⑨	896210
11	PAX-interacting protein 1	825640
12	unknown protein ⑦	808250
13	nascent polypeptide-associated complex subunit alpha, muscle-specific form isoform X3	788950
14	xanthine dehydrogenase	733740
15	EH domain-binding protein 1 isoform X1	484320
16	venom serine protease-like ②	470140
17	tubulin beta-1 chain isoform X1	411650
18	PREDICTED: nucleic-acid-binding protein from mobile element jockey	403840
19	general odorant-binding protein 1	390120
20	venom s1 protease 15	386140

Supplementary Table S3: Top 20 proteins in terms of the LFQ intensity value in gland extracts from *Arma custos* 5th-instar nymphs (GE-n).

Number	Protein	LFQ Intensity
1	uncharacterized protein LOC106684267 ①	50295000
2	E3 ubiquitin-protein ligase LRSAM1 isoform X2	34670000
3	venom serine protease-like ②	34063000
4	unknown protein ⑦	31407000
5	uncharacterized protein LOC106680269	27302000
6	unknown protein ⑨	24048000
7	venom serine protease-like ⑥	20118000
8	probable prefoldin subunit 4	15518000
9	signal-induced proliferation-associated 1-like protein 1	9128000
10	venom serine protease-like ⑤	6900600
11	protein turtle isoform X4	6630200
12	band 3 anion transport protein isoform X5	5443300
13	ATP-binding cassette sub-family F member 3	4750800
14	unknown protein ⑩	4512000
15	uncharacterized protein LOC106689219	3713600
16	transferrin-like	3667300
17	venom s1 protease 15	3580100
18	LOW QUALITY PROTEIN: insulin-degrading enzyme ②	3214400
19	cathepsin B-like cysteine proteinase 4	3193200
20	unknown protein ⑯	2977400

Supplementary Table S4: Top 20 proteins in terms of the LFQ intensity value in gland extracts from *Arma custos* adults (GE-a).

Number	Protein	LFQ Intensity
1	uncharacterized protein LOC106684267 ①	41060000
2	E3 ubiquitin-protein ligase LRSAM1 isoform X2	39493000
3	transferrin-like	17113000
4	myosin heavy chain, muscle isoform X22	16300000
5	unknown protein ⑦	6124600
6	LOW QUALITY PROTEIN: insulin-degrading enzyme ②	5471700
7	unknown protein ⑨	5459500
8	odorant-binding protein 18	5147900
9	uncharacterized protein LOC106680269	4714800
10	signal-induced proliferation-associated 1-like protein 1	3268900
11	apolipophorins ②	2604000
12	venom serine protease-like ⑥	2586000
13	venom serine protease-like ②	2312500
14	membrane-spanning 4-domains subfamily A member 14-like	2087800
15	trypsin precursor	1675500
16	ATP-binding cassette sub-family F member 3	1627100
17	band 3 anion transport protein isoform X5	1547600
18	venom s1 protease 15	1177600
19	venom serine protease-like ③	1111700
20	uncharacterized protein LOC106679174	841770

Supplementary Table S5: Top 20 proteins in terms of the LFQ intensity value in venom samples from *Arma custos* adults obtained by electrostimulation (VS-a).

Number	Protein	LFQ Intensity
1	unknown protein ⑦	40385000
2	uncharacterized protein LOC106684267 ②	34613000
3	uncharacterized protein LOC106684267 ①	25598000
4	uncharacterized protein LOC106680269	22537000
5	unknown protein ⑨	16498000
6	venom serine protease-like ②	16199000
7	venom serine protease-like ⑥	10299000
8	unknown protein ⑩	5581100
9	uncharacterized protein LOC106690607	4997500
10	myosin heavy chain, muscle isoform X22	4944900
11	uncharacterized protein LOC106689219	4918000
12	apolipophorins ②	4682600
13	puromycin-sensitive aminopeptidase isoform X1	4053000
14	cathepsin B-like cysteine proteinase 4	3554200
15	unknown protein ⑯	3280600
16	unknown protein ①	3177900
17	glutathione hydrolase 1 proenzyme-like isoform X1	3119000
18	uncharacterized protein LOC106679174	2887700
19	venom s1 protease 15	2808000
20	LOW QUALITY PROTEIN: insulin-degrading enzyme ②	2790400
